# There is no correlation between sublineages and drug resistance of *Mycobacterium tuberculosis* Beijing/W lineage clinical isolates in Xinjiang, China

**DOI:** 10.1017/S0950268814000582

**Published:** 2014-03-25

**Authors:** L. YUAN, Y. HUANG, L. G. MI, Y. X. LI, P. Z. LIU, J. ZHANG, H. Y. LIANG, F. LI, H. LI, S. Q. ZHANG, W. J. LI

**Affiliations:** 1 Department of Pathogenic Biology and Immunology, School of Medicine, Shihezi University, Shihezi, People's Republic of China; 2 The Third Division Hospital of Xinjiang Corps, Kashi, People's Republic of China; 3 The First Hospital of Shihezi University, Shihezi, People's Republic of China; 4 The People's Hospital of Changji State, Changji, People's Republic of China

**Keywords:** Beijing/W strains sublineages, drug resistance, *Mycobacterium tuberculosis*, Xinjiang province

## Abstract

The Beijing/W lineage strains are the major prevalent strains in China. The prevalence, mortality and drug-resistant rates of tuberculosis in Xinjiang, Northwestern China are higher than in other parts of the country. Our previous study results showed that the dominant strains of *Mycobacterium tuberculosis* (MTB) were ‘Beijing/W lineage’ MTB in Xinjiang; those strains had no significant correlation with drug resistance. We investigated whether the prevalence of ‘Beijing/W lineage’ sublineage strains was associated with drug resistance. We collected 478 sputum specimens from patients with pulmonary tuberculosis. Beijing/W strains and their sublineages were identified by distinguishing five specific large sequence polymorphisms, using polymerase chain reaction. All strains were subjected to a drug susceptibility test using the proportion method on Löwenstein–Jensen culture medium. In total, 379 clinical isolates of MTB were isolated and identified, 57·26% of these isolates were identified as Beijing/W strains, of which 11·06% isolates were in sublineage 105, 14·74% isolates in sublineage 207, 69·59% isolates in sublineage 181, and 4·61% isolates in sublineage 150. None of the isolates was in sublineage 142. Our data showed there were four sublineages of Beijing/W isolates in Xinjiang province, China. However, there were no correlations between drug resistance and the sublineages of Beijing/W strains.

## INTRODUCTION

The World Health Organization (WHO) estimates that currently more than one-third of the world's population is asymptomatically infected with tuberculosis (TB). China occupies second place behind India among the 22 countries considered to be high-burden countries that account for about 80% of new TB cases worldwide each year. Large sequence polymorphisms (LSPs) represent unique event polymorphisms that can be used to construct robust phylogenies for *Mycobacterium tuberculosis* (MTB) [1]. LSPs (robust phylogenetic markers) classify MTB into six major lineages, each of which is strongly associated with specific geographically distinct human populations [2].The East Asian lineage is one of these six lineages, and it includes the Beijing/W strains [3]. Beijing/W lineage strains were first identified in MTB isolates from the Beijing area of China after which it was named. These strains are defined by a spoligotype in which direct variable repeats 1–34 are missing [4]. Beijing/W lineage strains can also be recognized by several LSPs unique to Beijing/W lineage strains using genomic microarray approaches, which are further subdivided into five sublineages using specific regions of difference (RDs) [5]. One LSP (RD105 region) is 3467 bp in size and completely deletes Rv0072 and Rv0073 and truncates Rv0071 and Rv0074. The RD105 region serves as a useful marker for distinguishing Beijing/W lineage strains from non-Beijing/W lineage strains, because the RD105 region deletion was found in all Beijing/W lineage strains [6], but has not been found in any non-Beijing/W lineage strains [2]. RD142 (2851 bp), RD150 (2487 bp), and RD181 (711 bp) regions are variably deleted in Beijing/W lineage strains but are monophyletic for subdivisions of the Beijing/W lineage group [6].

Beijing/W lineage strains of MTB are endemically prevalent in eastern Asia [7], South Africa [8], and Russia [9]. Previous studies have revealed that Beijing/W lineage strains are associated with outbreaks [10] and treatment failures [11]. Studies of genetic relatedness suggest that Beijing/W strains also show evidence of clonal expansion [12], and the selective advantage of the Beijing clonal family may be an elevated mutation rate that facilitates the rapid accumulation of antibiotic resistance [13]. Beijing/W lineage strains are the major prevalent strains in China [14]. Xinjiang is a province in the northwestern area of China, and the prevalence, death rates and drug-resistant rates of TB are higher there than in other parts of the country. The WHO surveillance report on MTB drug resistance in Xinjiang showed the proportions of overall drug resistance, drug resistance in new cases, and re-treated smear-positive cases, were 26%, 26% and 31%, respectively, while the multidrug resistance (MDR) rate was 5% [15]. Therefore, the quality of TB control in this region clearly needs to be strengthened [15]. The main aim of this study was to classify the Beijing/W lineage strains of MTB in Xinjiang by identifying specific LSPs. We also sought to determine the drug susceptibility patterns of the sublineages of Beijing/W lineage strains and to determine whether the drug resistance of epidemic MTB is associated with the individual sublineages of Beijing/W lineage strains.

## MATERIALS AND METHODS

### Isolates selection

Sputum specimens (n = 478) were collected from pulmonary TB patients between June 2007 and June 2012 from Xinjiang province in China. The patients were all diagnosed as having pulmonary TB according to the national guidelines of China, and were treated in local lung disease hospitals or TB hospitals. Of these patients, 45 were from Akesu, 32 from Chang ji, 44 from Dushanzi, 32 from Hotan, 146 from Kashigar, nine from Kizilsu kirghiz, 14 from Manasi, 89 from Shihezi, 28 from Tumushuk, 25 from Tacheng, and 14 from Wusu. Following verbal and written consent, three sputum samples from each patient were collected at the same time. Information was obtained on sex, age, place of birth, recent smear-positive sputum tests, previous history of TB, and current address by a structured questionnaire. The study was performed with the approval of the Ethics Committee of the First Hospital of Shihezi University and performed in compliance with the Helsinki Declaration. Informed consent was obtained from all subjects before commencement of the study.

### MTB isolation and drug susceptibility test

Sputum samples were cultured on Löwenstein–Jensen (LJ) culture medium. The culture, identification and drug susceptibility tests of all strains were performed according to the TB diagnosis bacteriology test criteria of the China Antituberculosis Association [16] at the Ministry of Education Key Laboratory of Xinjiang Endemic and Ethnic Disease. Drug susceptibility testing was performed using the proportion method. Four first-line anti-TB drugs [isoniazid (INH), rifampicin (RFP), streptomycin (SM), ethambutol (EMB)] plus seven second-line anti-TB drugs [Ofloxacin (Ofx), Capreomycin (Cm), Kanamycin (Km), Amikacin (Am), P-aminosalicylic acid (PAS), Ethionamide (Eto) and Cycloserine (Cs)] were incorporated into LJ medium, at the following concentrations: 0·2 *μ*g/ml INH, 40·0 *μ*g/ml RFP, 4·0 *μ*g/ml SM, 2·0 *μ*g/ml EMB, 2·0 *μ*g/ml Ofx, 40·0 *μ*g/ml Cm, 30·0 *μ*g/ml Km, 40·0 *μ*g/ml Am, 1·0 *μ*g/ml PAS, 40·0 *μ*g/ml Eto, 40·0 *μ*g/ml Cs, respectively, and used to detect drug resistance. Isolates were scored as resistant to a specific drug when the growth rate was >1% compared to the control, or as sensitive when the growth rate was < 1% compared to the control as previously described [17].

### Genomic DNA extraction and molecular identification of MTB isolates

Mycobacterial genomic DNA was extracted from colonies growing on LJ medium. Scraped colonies were resuspended in 200–300 *μ*l distilled water and inactivated at 85°C for 30 min, before centrifugation at 5900 ***g*** for 5 min. The pellets were re-suspended in 300 *μ*l TE (pH 8·3), boiled for 30 min, and centrifuged at 9300 ***g*** for 5 min. Supernatants were collected and stored at –20°C until further use [18]. Molecular identification of the mycobacterial isolates was performed using polymerase chain reaction (PCR) amplification of the 16S rRNA and MTP40 genes [19]. The PCR mixtures consisted of 0·2 *μ*g DNA template, 3 *μ*l buffer, 4 *μ*l 10 mm DNTP, 1 *μ*l of each primer (10 pmol/*μ*l), and 1 *μ*l DNA *Taq* polymerase (2·5 U/*μ*l). The amplification cycle was 5 min at 95 °C; followed by 30 cycles of 40 s at 95°C, 50 s at 65°C, and 40 s at 72°C; with a final 10 min extension at 72°C. PCR products were analysed on a 2% agarose gel against a 100-bp DNA ladder.

### Identification of genomic deletions by PCR and multiplex PCR

PCR and multiplex PCR were performed to identify the sub-population structure of the MTB Beijing/W lineage strains. The RD105 LSP was deleted in all Beijing/W lineage strains and was able to be used as a marker to define the Beijing/W lineage strains [6]. A three-step PCR experiment was conducted to determine the presence or absence of the RD105 region. Primers were designed as described previously [20].

Further identification of the sublineages of MTB Beijing/W lineage strains were made on the basis of the variable appearance of the RD207, RD181, RD150, and RD142 deletions. In this study, we refer to sublineage 105 strains as those containing only deletions of RD105 region, while sublineage 207 strains also contained concurrent deletions of RD105 and RD207 regions, sublineage 181 strains had concurrent deletions of RD105, RD207 and RD181 regions, sublineage 150 strains had concurrent deletions of RD105, RD207, RD181, and RD150 regions, while sublineage 142 strains had concurrent deletions of RD105, RD207, RD181, and RD142 regions. PCR was used to identify sublineage RD181 and sublineage RD150, while multiplex PCR was used to identify sublineage RD207and sublineage RD142. Primers were designed as described previously [21] (Table 1). The amplification cycle was 4 min at 94°C; followed by 30 cycles of 45 s at 94°C, 45 s at 58°C, and 50 s at 72°C; with a final 10 min extension at 72°C. PCR products were analysed on a 1·5% agarose gel against a 100-bp DNA ladder. We randomly selected the PCR products of ten specimens from each sublineage for DNA sequencing.

**Table 1.** Primers used in large sequence polymorphisms (LSPs) and DNA sequence analysis for sublineages of *Mycobacterium tuberculosis* Beijing strains

LSP	Primer	Sequence (5′–3′)	Product size (bp)	
			Intact*	Deleted†
RD105	RD105-F	CGTGCACAGTTGGGTGTTTA	547	
	RD105-R	CGTCGTTTTCTGCCGATACT		283
RD207	RD207-F	GTCTGACGACTGACAGGGTG		
	RD207-R_int_‡	CACGATGGCCACCTCCATG	516	
	RD207-R_del_¶	CGTTGCGTGCTCGACGCTG		682
RD181	RD181-F	CAACAGCACCAGCATCGGAC	326	
	RD181-R	CTTGCTATCGGCGTCGTTGC		537
RD150	RD150-F	CCATCCTGGCGTTGGTTGG	505	
	RD150-R	CCGAGGACCTTACTGCGTG		300
RD142	RD142-F	GGAGGACACATGTCGCAACAC		
	RD142-R_int_	CGTGCAGCACGAACACCAC	400	
	RD142-R_del_	GTCGCAGCGCGAGTAGATC		577

* Intact = possessing an intact specific LSP region, e.g. RD105 region, RD207 region, etc.

† Deleted = specific LSP region was deleted.

‡ RD207-R_int_, Reverse primer of intact RD207 region by multiplex PCR.

¶ RD207-R_del_, Reverse primer of the deleted RD207 region by multiplex PCR.

### Statistical analysis

The statistical analyses were performed using the *χ*^2^ test and the Fisher's exact test.

## RESULTS

### Characteristics of patients and samples

**Fig. 1.**
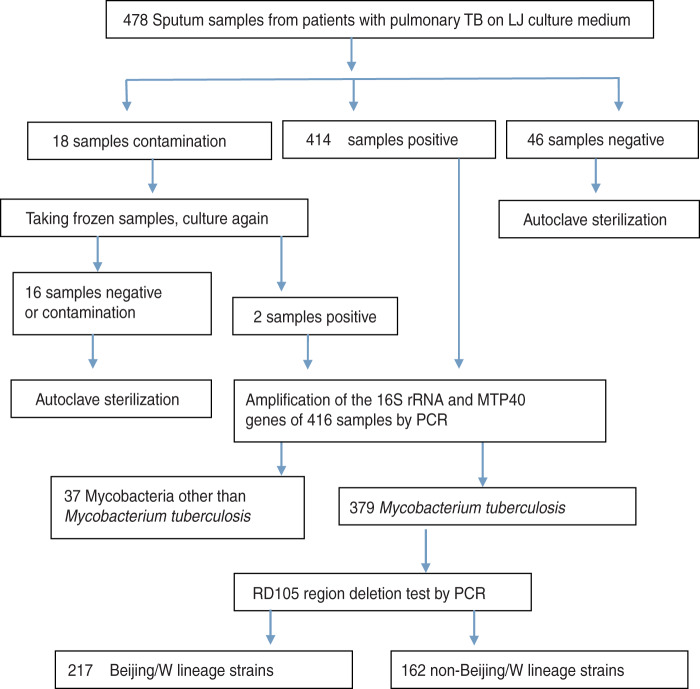
Flow diagram of numbers of eligible patients.

Among the 478 samples, 12·97% (62/478) of the specimens were either culture-negative or culture-contaminated and were therefore excluded, the remaining 87·03% (416/478) of the samples tested positive for mycobacterium. Of these, 8·89% (37/416) specimens tested positive for mycobacteria other than MTB and were excluded. Therefore, a total of 379 MTB culture-positive specimens were used for this study. A flow diagram shows the numbers of eligible patients, negative cultures, contamination, non-TB mycobacteria, and molecular tests (see Fig. 1).

### Use of RD deletions to differentiate between Beijing/W and non-Beijing/W lineage strains

A three-step PCR experiment was conducted to determine the presence or absence of RD105 region in the 379 isolates. Two hundred and seventeen isolates contained the RD105 region deletion and were therefore identified as Beijing/W lineage strains. These isolates were from 59·45% (129/217) males and 40·55% (88/217) females. One hundred and sixty-two isolates had no RD105 region deletion and were therefore identified as non-Beijing/W lineage strains (Fig. 1). Between Beijing/W lineage strains and non-Beijing/W lineage strains, there was no significant correlation with regard to gender by *χ*^2^ text (*χ*^2^ = 0·269, *P* = 0·604). Neither the Beijing/W lineage strains nor the non-Beijing/W lineage strains showed any significant correlation between new cases and re-treated cases by *χ*^2^ text (*χ*^2^ = 2·454, *P* = 0·117) (Table 2). Of 217 Beijing/W lineage strains, 85·71% (186/217) were from farmers, followed by workers (8·76%), students (3·23%), etc. (Table 2).

**Table 2.** Characteristics of patients infected by different sublineages of the Beijing strains

Groups …	105 (*n* = 24)	207 (*n* = 32)	181 (*n* = 151)	150 (*n* = 10)	
Characteristic					
Sex					
Female (*n* = 88)	9	9	66	4	
Male (*n* = 129)	15	23	85	6	*P* = 0·429
Treated cases and new cases					
New cases	7	12	52	3	
Retreated cases	17	20	99	7	*P* = 0·917
Age (years)					
0–24	3	5	16	1	
25–44	7	7	42	4	
45–64	10	13	46	3	
⩾65	4	7	47	2	*P* = 0·788*
Occupation					
Farmer (*n* = 186)	24	22	134	6	85·71%
Worker (*n* = 19)	3	4	10	2	8·76%
Administrator (*n* = 4)		1	3		1·84%
Student (*n* = 7)	1	2	4		3·23%
Teacher (*n* = 1)		1			0·5%
Beijing/W lineage strains (*n* = 217)			non-Beijing/W lineage strains (*n* = 162)		
Males (*n* = 129)	Females (*n* = 88)		Males (*n* = 92)	Females (*n* = 70)	*P* = 0·604
New TB cases (*n* = 74)	Re-treated cases (*n* = 143)		New TB cases (*n* = 68)	Re-treated cases (*n* = 94)	*P* = 0·117

* Value by Fisher's exact test.

### Drug susceptibility patterns of Beijing/W and non-Beijing/W lineage strains

We examined the distribution of drug resistance between Beijing/W lineage strains and non-Beijing/W lineage strains. Of the 217 Beijing/W lineage isolates, 26·73% (58/217) were resistant to at least one drug, and the overall resistance count included 49 isolates for first-line drug resistance and nine isolates for both first- and second-line drug resistance; 6·91% (15/217) of these were MDR-TB strains. Of the 162 non-Beijing/W lineage isolates, 24·69% (40/162) were resistant to at least one drug; 5·56% (9/162) of these were MDR-TB strains. There was no significant difference with regard to drug resistance between Beijing/W lineage strains and non-Beijing/W lineage strains (*χ*^2^ = 0·201, *P* = 0·654) and MDR-TB (*χ*^2^ = 0·119, *P* = 0·731). Two, two, one, and one ‘Beijing/W lineage’ sublineage strains were resistant to Ofx, Cm, Am, and Km respectively; while three, three, and one ‘Beijing/W lineage’ sublineage strains were resistant to PAS, Eto and Cs, respectively. Because the numbers of ‘Beijing/W lineage’ sublineage strains for drug resistance to seven second-line anti-TB drugs is too few, we did not perform statistical analyses for those drug-resistant strains (Table 3).

**Table 3.** Distribution of drug resistant strains in different sublineages of the Beijing strains

Groups …	105 (*n*=24)	207 (*n*=32)	181 (*n*=151)	150 (*n*=10)	*P*
Resistant to:					
INH (*n* = 33)	2	4	27	0	0·402*
RFP (*n* = 16)	1	2	13	0	0·961*
SM (*n* = 32)	2	5	25	0	0·549*
EMB (*n* = 15)	1	1	13	0	0·743*
MDR-TB (*n* = 15)	1	1	13	0	0·743*
Ofx (*n* = 2)			2		
Cm (*n* = 2)		1	1		
Am (*n* = 1)				1	
Km (*n* = 1)				1	
PAS (*n* = 3)			3		
Eto (*n* = 3)	1	1		1	
Cs (*n* = 1)		1			

* Value by Fisher's exact test.

### Characteristics of MTB isolates in different sublineages

Of the 217 Beijing/W isolates, 11·06% (24/217) had only the RD105 region deletion and were identified as sublineage 105. A group with concurrent deletions of RD105 and RD207 regions was identified as sublineage 207 (*n* = 32, 14·75%), a group with concurrent deletions of RD105, RD207 and RD181 regions was identified as sublineage 181 (*n* = 151, 69·59%), and a group with concurrent deletions of RD105, RD207, RD181, and RD150 regions was identified as sublineage 150 (*n* = 10, 4·60%). PCR verification did not detect the RD142 region deletion in any of the 217 isolates. We therefore concluded that none of the clinical isolates was in sublineage 142 in our specimens. The age, gender, history of treatment and the proportion of resistance to any drug did not show statistically significant correlations to any of the sublineages (Table 2).

## DISCUSSION

Large chromosomal deletions, also termed LSPs, are unique event polymorphisms [1]. In recent years, these polymorphisms have been widely used to research the phylogenies of MTB. Whether or not the bacterial lineage influences the development of the disease has been investigated by researchers for many years; however, this still remains unclear. Some studies have shown that the Beijing/W lineage of clinically important MTB strains has led to outbreaks of TB [9, 22, 23] and been associated with drug resistance [24, 25]. However, it is the significance of this association that varies in different countries [9, 26]. This may be due to the different proportions of the sublineages of Beijing/W lineage strains present in different local populations [27–29].

In our study, we examined the frequency of occurrence of each sublineage and drug resistance of Beijing/W lineage strains in Xinjiang, China. MTB isolates were found to be subdivided into four sublineages using specific LSPs as markers. There was no isolate with the RD142 region deletion in our study. Similar reports have shown that 85 isolates from Vietnam [30], 73 isolates from Tuscany, Italy [31], 28 isolates from Myanmar [32], and 14 isolates from Sri Lanka [33] also did not have the RD142 region deletion. However, a study from Beijing in China, found that 26·42% (79/299) of strains of the Beijing/W lineage belonged to sublineage 142 [34], and another study in Arkansas, USA, found that 13/39 (33%) strains of the Beijing/W lineage belonged to sublineage 142 [35].

Sublineage 142 is one of the more puzzling sublineages of the Beijing/W lineage of MTB. Since there are low isolate numbers in sublineage 142, some scholars have conjectured that it may be the most recently evolved of the sublineages in this monophyletic group and has therefore not yet had time to proliferate and disseminate. It may therefore be less pathogenic [36]. However, another report showed that 80% of the strains from sublineage 142 (8/10 tested) synthesized detectable quantities of phenolic glycolipid (PGL-tb), which is an important virulence factor for a fraction of the Beijing/W lineage isolates that are likely to be associated with pulmonary TB manifestations within the human host [37]. PGL-tb suppresses the release of the pro-inflammatory cytokine, tumour necrosis factor-alpha, as well as interleukins 6 and 12 *in vitro* [38], and sublineage 142 has been associated with extra-pulmonary TB [37]. These findings imply that sublineage 142 may in fact be more pathogenic than previously thought. Sublineage 150 is another interesting sublineage of the Beijing/W lineage and is worthy of discussion. Two recent reports have shown that the numbers of sublineage 150 have occupied second place, behind sublineage 181, among the top five sublineages of Beijing/W lineage [21, 36]. Sixty percent of the strains from sublineage 150 (6/10 tested) synthesized PGL-tb [37] and sublineage 150 was also associated with extra-pulmonary TB [37]. In 2007, Hanekom *et al*. used IS*6110* DNA fingerprinting, single-nucleotide polymorphisms (SNPs) and LSPs to analyse the genomic structure of the Beijing/W lineage, which suggested that sublineage 150 has the ability to transmit; sublineage 150 has also been associated with MDR-TB in rural settings [27]. The most obvious characteristic of sublineage 181 is the number of strains accounted for absolute advantage in Beijing/W lineage MTB [21, 36]. In our study, 69·59% of isolates belonged to sublineage 181, this result is similar to current reports in San Francisco (sublineage 181: 64·4%) [36]. The 88·74% (134/151) of sublineage 181 in Xinjiang were farmers from 11 different regions. Based on these patients’ current address information, we did not find the means of mutual transmission and direct contact. Therefore, these pulmonary TB patients of sublineage 181 referred to sporadic cases. A study from Reed *et al*. found that 10% of the sublineage 181 strains tested produced PGL-tb [37]. There are only a few reports on sublineage 207, which is significantly associated with the highest frequency of secondary cases [36]. Reed *et al*. reported there was only one strain of sublineage 207, and found that it did not produce PGL-tb [37]. Previous studies did not find any associations between drug resistance and pathogenicity for sublineage 105 [27].

Some studies have shown that Beijing/W lineage strains are associated with drug resistance and MDR [9, 39]. MDR is significantly higher in Beijing/W lineage strains compared to non-Beijing/W lineage strains [40]. To further discuss the association between Beijing/W lineage strains and drug resistance, some scholars have subdivided the Beijing/W lineage strains into sublineages using different molecular epidemiological methods and studied the association between these sublineages and drug resistance. Previous studies have shown that several sublineages occur with a significantly higher frequency in the MDR/XDR (extensively drug-resistant) population [9]. These findings suggest that different sublineages of the Beijing family may differ in their mechanisms of adaptation to drug selection pressures. In these studies, the Beijing/W lineage strains were not subdivided into sublineages based only on LSPs, but synonymous SNPs and IS*6110* DNA fingerprints were also used at the same time [9, 34]. Other studies have shown that the Beijing/W lineage can be roughly subdivided into ancient sublineages [possessing an intact narcotics task force (NTF) region] and modern sublineages (possessing one or two IS*6110* insertions on the right side of the NTF region) by determining whether or not the NTF region is present within the IS*6110* sequence [28].

There are only a few reports that analyse the subdivisions of the Beijing/W lineage using LSPs and discuss the association between these Beijing/W lineage and drug resistance [36]. For the low frequency of drug-resistant TB in San Francisco, there are no statistical correlations between drug resistance and the different sublineages. Although our results revealed a high frequency of drug-resistant MTB in Xinjiang, our data still did not demonstrate any association between drug resistance and the different sublineages. The reason for this may because the sample sizes for sublineages 150 and 142 (those most likely to be related to drug resistance and pathogenicity) were too small. The Beijing/W lineage stains have previously been closely associated with nosocomial infections and community outbreaks, global transmission, and drug resistance. Xinjiang is one of the drug-resistant TB hotspots in China, thus the increased drug-resistance rate of MTB remains a serious problem in Xinjiang. Further research using a larger sample of isolates is required to fully explore this association between drug resistance and the different Beijing/W lineage sublineages. Given that no correlation has been found between drug resistance and sublineages, it should be interesting to use molecular epidemiological methods to distinguish individual isolates in order to identify potential outbreaks of drug-resistant isolates.
